# Frequency and Demographic Profile of Odontogenic Cysts in Riyadh, Saudi Arabia: Retrospective Multicenter Study

**DOI:** 10.3390/diagnostics13030355

**Published:** 2023-01-18

**Authors:** Asma Almazyad, Marzouq Almutairi, Nasser Almadan, Mohammed Alamro, Fahad Maki, Turki S. AlQuwayz, Assem S. Alrumeh

**Affiliations:** 1 College of Dentistry, King Saud bin Abdulaziz University for Health Sciences, P.O. Box 3660, Riyadh 11481, Saudi Arabia; 2 King Abdullah International Medical Research Center, P.O. Box 3660, Riyadh 11481, Saudi Arabia; 3 Department of Pathology and Laboratory Medicine, King Abdulaziz Medical City, Ministry of National Guard Health Affair, P.O. Box 22490, Riyadh 11426, Saudi Arabia; 4 Prince Sultan Military Medical Center, P.O. Box 7897, Riyadh 12233, Saudi Arabia

**Keywords:** odontogenic cysts, tertiary center, apical radicular cyst, dentigerous cyst, odontogenic keratocyst, glandular odontogenic cyst, biopsy, pathology

## Abstract

Odontogenic cysts (OCs) are etiologically diverse conditions with a shared origin in the jaws. Unfortunately, there is a scarcity of published data regarding OC frequency, treatment, and follow-up information in Saudi Arabia, especially from tertiary centers. Objectives: The study aims to assess the relative frequency, clinicopathological features, treatment, and follow-up of OCs in three tertiary medical centers. Methods and Material: OCs were identified from King Abdulaziz Medical City, King Fahad Medical City, and Prince Sultan Military Medical City from January 2010 to December 2021. Results: Three hundred and seventy-two cases of OCs were identified from the archive of three medical centers in Riyadh; one hundred and forty-nine (40%) cases were retrieved from Prince Sultan Military Medical City. The mean age of all OCs was 32 years (range 2–90), with 225 (60.4%) cases occurring in males. There was an almost equal distribution of OCs between the maxilla (47.0%) and the mandible (53.0%). The apical radicular cyst (ARC) accounted for half of the cases, followed by dentigerous cyst (DC) (29.3%) and odontogenic keratocyst (OKC) (14.2%). Enucleation was the most common treatment modality (52.8%), followed by excision (35.0%). Thirteen cases showed recurrence: one ARC, four DCs, and eight OKCs. Conclusion: This is the first large multicenter study of OCs in Riyadh, Saudi Arabia. All three centers showed that ARC was the most common, followed by DC and OKC.

## 1. Introduction

Odontogenic cysts (OCs) are an uncommon diverse group of pathologies that exclusively occur in the oral cavity, mainly in the jawbones and less frequently in the gingival tissues. They mainly arise from the remnants of tooth development (odontogenesis), such as dental lamina and the rests of Malassez [1]. OCs are mainly divided into inflammatory and developmental cysts. The most recent WHO classification of odontogenic and maxillofacial bone tumors (5th edition) published in 2022 included two inflammatory and seven developmental cysts. Few of these OCs have clinical subtypes mentioned and reviewed in Table 1 [2].

The primary changes in the 2022 classification are the diagnostic criteria for a calcifying odontogenic cyst (COC) and glandular odontogenic cysts (GOC). The presence of ghost cells that may undergo calcification is sufficient for COC diagnosis, while the presence of the ameloblastic epithelial lining is no longer required. Additionally, the presence of odontoma with COC is no longer considered a separate subcategory of COC. Flower et al. [3] reviewed and identified ten histological features of GOC and suggested the presence of seven is strongly suggestive of GOC and was previously adopted by the WHO [4]. However, seven out of ten diagnostic criteria to favor GOC diagnosis is dropped from the current classification, with the presence of hob-nail cells as the sole criterion essential for GOC [2]. OKC was previously classified as a keratocystic odontogenic tumor due to its aggressive behavior. However, experts agreed the current evidence was insufficient then and reclassified them into a cyst in the WHO 2017 Classification [4]. A recent report showed 93% of sporadic odontogenic keratocysts (OKC) demonstrate *PTCH1* mutation. However, OKC remains in the cyst category in the 2022 classification [2,5].

Several papers and systematic reviews reported the prevalence and frequency of OCs in oral biopsy services worldwide, ranging from 6.8–17.2% of all biopsies [6,7,8]. Moreover, one study examined the prevalence of OCs in a single oral pathology laboratory at the College of Dentistry, King Saud University, Riyadh, Saudi Arabia, and reviewed 470 cases of OCs and found that apical radicular cyst (ARC) was the most common, followed by dentigerous cyst (DC), consistent with the previous literature [9]. However, whether this is true for OCs in an anatomical pathology laboratory at tertiary hospitals still needs to be determined. To our knowledge, no studies assessed the treatment modalities and outcomes of OCs in the Saudi population. Therefore, this paper examines the relative frequency of OCs in three tertiary medical centers. In addition, the current study assesses different treatment modalities and follow-up information on OCs.

## 2. Method and Materials

The current study is a retrospective analysis of patients diagnosed with OCs from the archives of three tertiary medical centers: King Abdulaziz Medical City (KAMC), King Fahad Medical City (KFMC), and Prince Sultan Military Medical City (PSMMC), Riyadh, Saudi Arabia from January 2010 to December 2021. All study procedures were performed according to the Declaration of Helsinki. Institutional review board approval was obtained from King Abdullah International Medical Research Center (IRB# NRC21R/222/06) and King Fahad Medical City (IRB# 00010471).

**Figure 1:**
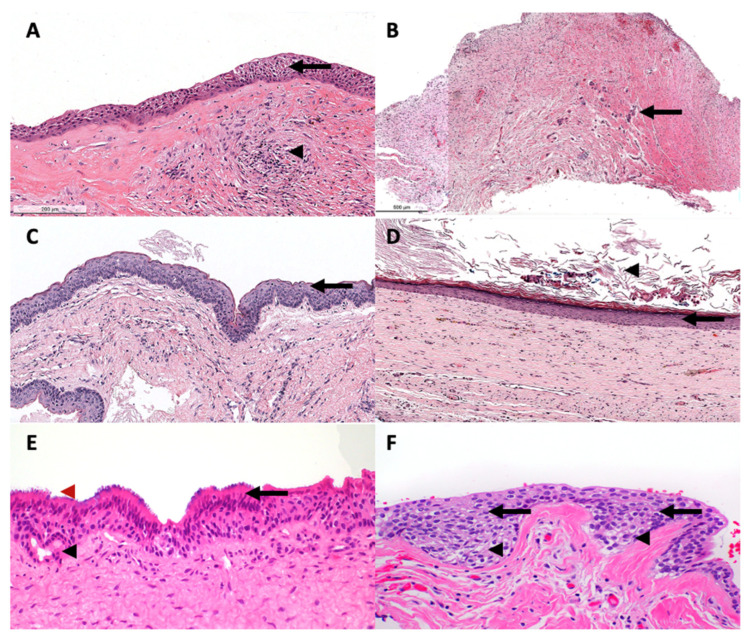
A representative histological photomics of developmental OCs. (**A**) DC is lined by uniformly thin nonkeratinized squamous cell epithelium (arrow) with fibrovascular wall and scattered inflammatory cells (arrowhead). EC show similar histological features to DC; however, located in the lamina propria (**B**) Hyperplastic dental follicle consists of delicate fibrocollegenous stroma with scattered odontogenic epithelial islands (arrow). (**C**) OKC is lined by uniformly thin parakeratinzed squamous cell epithelium with surface corrugation and hyperchromatic basal cell layer (arrow). (**D**) OOC is lined by uniformly thin orthokeratinized stratified squamous epithelium (arrow) with keratin debris within the cyst lumen (arrowhead). (**E**) GOC is lined by uniformly thin nonkeratinized squamous epithelium with abluminal eosinophilic cuboidal (hobnail) cells (arrow) with ducts (black arrowhead) and cilia (red arrowhead) observed in the cyst lining. (**F**) LPC is lined by uniformly thin nonkeratinized squamous epithelium-exhibiting clear cells (arrowhead) and epithelial plaques (arrow).

Demographic and clinical data were extracted from medical records, including age, gender, location, treatment, and follow-up information. In addition, hematoxylin and eosin-stained slides of all the cases were reviewed by a certified oral pathologist (AA) to confirm the histopathologic diagnosis based on the 2022 WHO classification of odontogenic and maxillofacial bone tumors. When in doubt or when OCs showed significant inflammation masking the histological features, radiographic features were evaluated to reach a final diagnosis. Histological features are described in Table 2, and representative histopathological images of each OC are illustrated in Figure 1.

The inclusion criteria are as follows:

1. Patients with histopathological features compatible with the diagnosis of OCs.
2. Patients with available demographic, clinical, pathological reports, and histological slides.

The exclusion criteria are as follows:

1. Patients with incomplete clinical data.
2. Patients with missing pathological specimens.

Descriptive analysis was conducted for the patient’s age, gender, location, OC type, treatment, and follow-up using STATA 14.2 software (StataCorp., Taxes, USA). When applicable, the correlation between different OCs to other variables was analyzed using the Chi-squared test for categorical variables and ANOVA for continuous variables. A *p*-value less than 0.05 was considered statistically significant. Graphs were generated using STATA 14.2 software (StataCorp., Taxes, USA) and GraphPad (Prism 9 software).

## 3. Results

**Figure 2:**
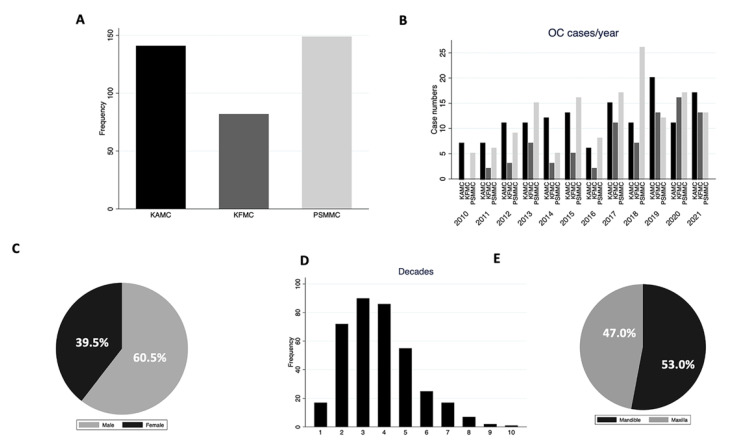
(**A**) OC distribution at each center. (**B**) Distribution of OCs at each center per year. PSMCC had the highest frequency in 2018. (**C**) Gender distribution of OCs. (**D**) Age distribution of OCs (**E**) Location distribution of OCs.

**Figure 3:**
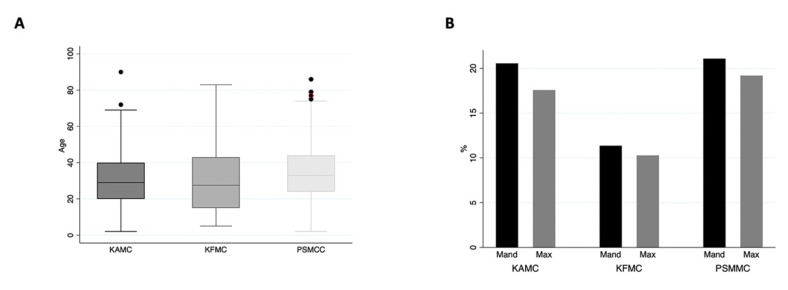
(**A**) The mean age of OCs at each center is relatively similar, with a *p*-value of 0.1. (**B**) Location distribution of OCs at each center.

Three hundred and seventy-two cysts were found in the archives of the three different centers in Riyadh, Saudi Arabia, for 12 years. One hundred and forty-nine (40.0%) and one hundred and forty-one (38.0%) cases were retrieved from PSMMC and KAMC, respectively. Only eighty-two (22.0%) cases were from KFMC (Figure 2A). Figure 2B illustrates the number of cases per year at each center. There has been an increase in OC diagnosis since 2017, with the highest cases seen in 2018 at PSMMC. A total of 225 (60.5%) cases were males and the male-to-female ratio was 1.53:1, with a lesser ratio in the KAMC and KFMAC cohorts and a higher ratio in the PSMMC cohort (Figure 2C and Table 3). The mean age of all cases was 32 years, with a wide age range from 2 to 90 years. However, eighty-one patients were reported in the second to fifth decades (Figure 2D). There was no significant difference in the age means between the three centers with a *p*-value of 0.1 (Table 3 and Figure 3A). There was a similar mandible-to-maxilla distribution of OC, with 196 (53.0%) in the mandible (Figure 2E). A similar location distribution was seen at each center (Figure 3B and Table 3).

**Figure 4:**
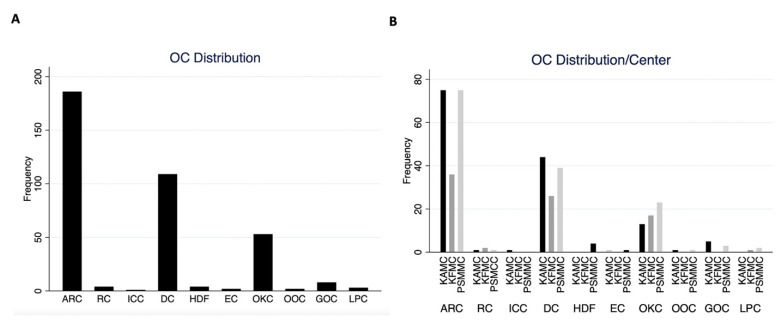
(**A**) Overall OC frequency in the current cohort. (**B**) OC frequency in each center.

Half of the cases reviewed at all centers were ARC (50.0%). DC was the second most common odontogenic cyst (29.3%), followed by OKC (14.2%) at all three centers (Figure 4A). Similar OCs distribution was observed at each center (Figure 4B). Eight cases of GOCs were retrieved from three centers. Only one ICC was reported in this cohort found in KAMC.

Table 4 summarize the clinicopathological feature of each OC. Treatment information was available for 352 patients; in general, the most common treatment was enucleation without curettage (52.8%), an excision (35.0%), or, less likely, enucleation with curettage (6.8%) (Table 5). Follow-up information was available for 320 cases, and only 13 (4.0%) showed recurrence (Table 6).

**Figure 5:**
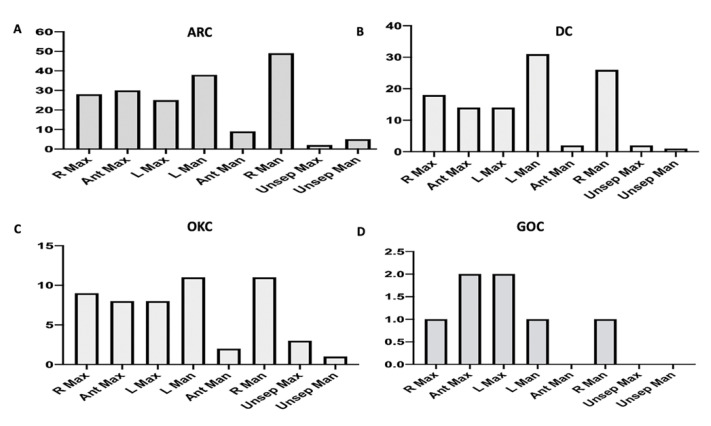
Distribution of OCs according to the specific location. (**A**) ARC, (**B**) DC, (**C**) OKC, (**D**) GOC. (Abbreviations: R Max, right maxilla; Ant Max, anterior maxilla; L Max, left maxilla; L Man, left mandible; Ant Man, Anterior mandible; R Man, right mandible; Unsep Max, unspecified location in maxilla; Unsep Man, unspecified location in mandible.

There were 186 (50.0%) ARCs with a mean age of 34.2, and 102 (54.8%) were males. The most common location was the mandible (54.31%), most commonly in the posterior mandible (Figure 5). The primary treatment modality used for ARC was enucleation without curettage (56.4%), an excision (35.5%), or, less likely, enucleation with curettage (7.0%) (Table 5). One hundred fifty-nine cases had follow-up information, and only one patient showed recurrence (Table 6).

There were 108 (29.3%) cases of DC with a mean age of 26, with 72 (66.1%) being males. Similar to ARC, DC’s most common specific location was the mandible (64.2%) and mainly in the posterior region (Figure 5). For DC, the primary treatment modalities were enucleation without curettage (60.4%) and excision (32.1%), with only two cases treated with marsupialization (Table 5). Ninety-two patients of DC were followed-up after treatment, and only four (4.3%) patients showed recurrence (Table 6). 

OKC was the third most common OCs found at the three centers, with 53 (14.2%) cases. The mean age was 34, and 38 (71.7%) patients were males. Almost two-thirds of the cases were in the mandible (73.6%) and, more specifically, the posterior mandible (Figure 5). Enucleation without curettage (38.5%) was the most common treatment of OKC, followed by excision (32.7%), and only 12 (23.1%) cases were treated by curettage. Two cases were treated with resection and one case with marsupialization (Table 5). Follow-up information was available for forty-seven, and eight (17.0%) patients showed recurrence (Table 6). Additionally, four patients presented with multiple OKCs and were diagnosed with Nevoid basal cell carcinoma (Gorlin) syndrome.

There were only eight cases of GOC retrieved from KAMC and PSMMC. The mean age of GOC patients was 40 and showed an equal gender distribution. Furthermore, most cases were in the maxilla, mainly the posterior region (Figure 5). Three GOCs were excised, two were enucleated only, and two were enucleated and curetted. Only one patient underwent bone resection (Table 5). None of the patients showed recurrence after follow-up. Other cysts were low in frequency to make any demographic inference (Table 4). Additionally, there was no report of COC, BOC, or gingival cysts in the adults in this cohort. 

## 4. Discussion

This is a retrospective study reviewing 372 cases of OCs, including demographic data (age and gender), OCs location, OC type, treatment, and follow-up information. This study is considered the 2nd largest series of OCs in the Gulf countries. Although there are three studies that examined the frequency of OCs in the gulf region (Saudi Arabia, Kuwait, and the United Arab Emirates (UAE)) (Table 7) [9,10,11], these studies are based solely on a single pathology service, while our research retrieved cases from three major tertiary centers serving Riyadh, Saudi Arabia; hence, it provide a deeper insight into the frequency of OCs in our region. Additionally, the current study reviewed treatment options and available follow-up information on OCs, which was not discussed previously. The frequency of OCs in our centers is very low. It ranges from 0.1–0.2% of all cases submitted in anatomical pathology laboratory, confirming the rarity of these conditions compared to other pathologies occurring in other organs. The reported range in the literature is from 6.8–17.2% of all biopsies; this is considered high in volume compared to the one reported in the current study. This is because cases in our series were retrieved from anatomical pathology laboratories that receive biopsies and resections from all body organs. In contrast, oral pathology services only focus on diagnosing maxillofacial pathologies.” 

There was variability in the duration reported in the three gulf studies ranging from 5 years to 20 years, which makes it difficult to compare the results of our study with the previous ones (Table 7). However, the current study showed similar age mean to Alsheddi et al. [9]. The other two failed to mention their cohort mean age. All studies show OCs are more common in males, but in the study from the UAE, OCs were more common in females [10]. There was a similar equal mandible to maxilla distribution of OCs in all studies except for the paper published in Kuwait, where the OCs were more common in the mandible [11]. Despite the difference in these studies, all showed that ARC is the most common cyst, followed by DC and OKC. It is worth mentioning that the Al-Rawi et al. [10] series did not report any OKCs. Few cases of GOCs were reported by Alsheddi et al. [9] and Ali M A et al. [11]; however, we had the highest number of GOC cases. Although this is a rare cyst, we speculate that few GOCs were included in the DC category since they share similar clinical and histological features. 

The most common cyst in our series was ARC accounting for 50.0% of all cases. The frequency was similar to those reported in Chile, Brazil, France, and Turkey [12,13,14,15], while others mentioned a higher range of 65% to 84%, such as in Canada and Italy (Table 8) [16,17]. The higher percentage in these reports is most likely skewed due to variability in diagnostic criteria used for OCs and study sample size in the aforementioned series. However, all previous studies showed higher male predilection to ARC, mainly in the third to fourth decades, similar to the current research. Most of the studies showed mandibular preference with a comparable ratio to our report, except for Tamiolakis et al. [7] and Jones et al. [6], where ARC occurred more in the maxilla. ARC is typically treated by non-invasive endodontic therapy of the offending tooth [18]. However, submitted ARCs to the pathology laboratory are usually enucleated with or without curettage, or excised. It rarely recurs unless the treated tooth was reinfected, and only patients in our series showed recurrence.

ICC, or inflammatory paradental cyst or mandibular bifurcation cyst, is an inflammatory odontogenic cyst that arises on the distobuccal aspect of mandibular third molars and is less likely on the buccal aspect of partially erupted first or second molars. It occurs concurrently with pericoronitis and occasionally due to the presence of enamel pearls in the buccal surface of the tooth [19]. Only one case of ICC was found in our series and was treated with marsupialization. Alsheddi et al. [9] only reported five cases, while other gulf countries reported none [10,11]. Although ICC is a rare cyst, we assume it is under-reported, since most cases are treated conservatively or go undetected and resolve spontaneously with tooth eruption. On the contrary, Tamiolakis et al. [7] and Avelar et al. [13] reported a more significant number of ICC, ranging from 28 to 57 cases. One explanation for the higher number of cases in their series is the larger sample size, which includes a young population. On the other hand, the residual cyst is another rare inflammatory odontogenic cyst seen when ARCs are left behind after tooth extraction. We have found only four (1.1%) cases with male predilection, an average age of 43 years, and equal jaw distribution. The frequency of residual cysts was low in most of the published literature, with a range of 0.6% to 8.0% and a wide age range spanning the second to eighth decade of life [8,20]. However, it always showed mandibular preference consistent with the ARC location preference [7].

The second most common cyst in the current series was DC (29.3%), a developmental odontogenic cyst that arises from epithelial metaplasia of reduced enamel epithelium of impacted teeth [21]. DC was also the second most common OC in most of the published series, with frequency ranges from 11.3% to 35% with male predilection and a mean age of 26 years [1,8,15,22]. DC was more frequent in the mandible; there was no difference observed in our cohort [23]. Additionally, the majority of cases were enucleated or excised, and only four cases showed recurrence. Two of the four recurred cases were enucleated without curettage, and two were treated with enucleation and curettage. DC recurrence is rare and not well documented in the literature [24]. EC is the soft tissue analogue of DC and is typically associated with unerupted primary central incisors or permanent first molar. Our series reported two cases related to primary teeth in the mandible in the first decade. EC is usually resolved spontaneously in 40% of cases if left untreated upon tooth eruption. As such, it is rarely biopsied and sent to the pathology service [25]. 

OKC was the third most common cyst, accounting for 14.25% of the cases. OKC is a locally aggressive development odontogenic cyst with a high recurrence rate. OKC generated a lot of debate on the nature of the cyst. It was considered a tumor and named keratocystic odontogenic tumor in WHO 2005. Later, the odontogenic keratocystic tumor was reclassified as a cyst in WHO 2017 and remained a cyst in the 2022 classification. Although it was classified as a tumor for almost 13 years and was included in the odontogenic tumor frequency, most of the prevalence and frequency studies of OCs had OKC ranging from 1.2% to 20% [14,16,17]. One study from Chile showed a similar frequency of our series (14.3%) [12], while a systematic review of OCs globally in 2013 showed a relatively close frequency (11.7%) [1]. Demographic data in the current study and previously published studies are consistent; OKC occurred more commonly in males in the second to fourth decades with mandibular preference with a male-to-female ratio range from 2:1 to 4:1 [14,17]. Patients with multiple OKCs should be evaluated for Gorlin (Nevoid basal cell carcinoma) syndrome. Gorlin syndrome patients exhibit palmer and planter pits, calcified flax cerebri, bifid ribs, and other features. Additionally, they have the risk of developing multiple basal cell carcinoma within the first two decades [26]. Interestingly, we had four patients with multiple OKC and diagnosed with Gorlin syndrome; other Gulf countries studies did not detect any Gorlin syndrome patients [9,10,11]. This is most likely due to the nature of the service. Our cohort was treated at tertiary centers with multi-disciplinary teams where patients are treated comprehensively and are more likely to diagnose other symptoms of this syndrome. There is abundant literature on OKC treatment, including enucleation, marsupialization, and resection [27,28]. Other adjunctive therapies that reduce OKC recurrence include cryotherapy with liquid nitrogen or Carnoy’s solution application after cyst enucleation [29]. However, most of the cysts in the series were treated with enucleation or cyst excision without any adjunctive therapy. There were 8 (17.0%) OKCs showed recurrence that was treated with enucleation; this treatment modality showed the highest recurrence rate up to 50% in the literature [30].

Only 8 (2.1%) cases of GOC, a rare developmental cyst, showed glandular differentiation. GOC is classically present in the anterior mandible crossing the middle, while our series showed seven cases were in the maxilla [31]. We reviewed the histological slides to confirm the diagnosis and showed that these cases are not DC showing duct formation or mucous metaplasia. GOC is an aggressive cyst with a recurrence rate of up to 20.0% [31]; however, none of the GOC in this report showed recurrence with a short follow-up period ranging from 1–4 years. We did not detect any gingival cysts in our series, a rarity, and the frequency in other reports ranges from 0.4% to 2.6% [12,32]. Additionally, COC was previously classified as a tumor in WHO classification and reported as such in the literature. Few series reported COC in frequency studies of OC, ranging from 0.3% to 1.3% [6,32]. However, COC was not reported in this series. 

The nature of our study has a few drawbacks. Mainly, retrospective studies occasionally fail to capture all subjects at the centers involved in the study. There is always missing information, especially regarding location and loss of follow-up. About 9% of the patients in our series were lost in follow-up. Additionally, the study by Alsheddi et al. and our own are both focused on the frequency of OCs in Riyadh, Saudi Arabia. In contrast, the frequency of OCs in other regions in Saudi Arabia remains unknown. 

## 5. Conclusions

OCs are a rare group of conditions unique to the jaws, which arise from tooth development. The frequency of OCs has been heavily studied in other countries. However, this is the first multicenter study in Saudi Arabia and the gulf countries to shed light on these rare entities and compare them to others. Half of the OCs found in this cohort were inflammatory in origin. Clinicopathological features were consistent with the previous report, except that GOC was more common in the maxilla. A total of 7.5% of OKC patients were diagnosed with Gorlin syndrome. More studies in other Saudi regions, such as Jeddah, Dammam, and Jizan, will help determine the OC frequency accurately in the Saudi population.

## Figures and Tables

**Table 1:** The 5th edition of WHO classification of odontogenic cysts.

Inflammatory Cysts	Abbreviation	Definition	Clinical Presentation	Radiographic Presentation
Radicular cyst (Apical) *	ARC	Inflammatory cyst associated with nonvital tooth.	Asymptomatic or swelling with history of pain or abscess.	Well-demarcated radiolucency associated with the apex of necrotic tooth.
Residual Cyst	RC	ARC remained after tooth extraction.	Incidental finding in radiograph.	Round to oval radiolucency at the site of previous tooth extraction.
Inflammatory collateral cyst	ICC	Buccal to roots of partially or recently erupted permanent molars.	Pericoronitis or asymptomatic swelling.	Well-demarcated radiolucency located buccally to the tooth.
**Developmental cysts**				
Dentigerous cyst ^+^	DC	Developmental cyst surrounding the crown of unerupted tooth.	Asymptomatic cyst frequently discovered when the tooth fails to erupt, most commonly in the third molars or canine.	Well-demarcated radiolucency associated with impacted tooth and attach to the cemento-enamel junction.
Hyperplastic dental follicle	HDF	Pericoronal radiolucency associated with an impacted tooth.		
Eruption cyst	EC	Superficial EC occurs in the soft tissue.	Bluish to reddish nodule covering an unerupted permanent central incisor or first molar.	N/A
Odontogenic keratocyst	OKC	Aggressive parakeratinzed OC with high recurrence rate.	Asymptomatic swelling commonly occurs in the posterior mandible.	Well-demarcated unilocular or multilocular radiolucency with anterior-posterior expansion, 35% associated with an unerupted tooth.
Orthokeratinized odontogenic cyst	OOC	Rare, orthokeratinized OC with insignificant recurrence rate.	Asymptomatic swelling and many found incidentally.	Well-demarcated unilocular radiolucency, 70% associated with an impacted tooth.
Glandular odontogenic cyst	GOC	Rare, aggressive OC showing glandular differentiation.	Slowly expansile swelling, most commonly in the mandible with anterior preference.	Well-demarcated unilocular or multilocular radiolucency that shows tooth displacement and root resorption. Lesions tend to reach a large size and cross the midline of the mandible.
Calcifying odontogenic cyst	COC	Rare OC shows ghost cells that tend to calcify and are commonly seen with odontoma.	Asymptomatic lesions with equal mandible to maxilla preference.	Well-demarcated unilocular mixed radiopaque lesion, one-third of the cases are radiolucency.
Lateral periodontal cyst and Botryoid odontogenic cyst	LPC/BOC	Rare OC shows non-keratinized epithelium with epithelial plaques.	Asymptomatic cyst between mandibular canine and premolar alveolar bone.	Pear-shaped radiolucency between mandibular canine and premolars, BOC is a variant of LPC showing multilocular radiolucency with a high recurrence rate.
Gingival cyst of adult	GCA	Soft tissue analogue of LPC, most commonly located in the mandibular attached gingiva.	Painless discrete bluish and translucent nodule between canine and premolars.	N/A

* RC is a clinical variant of ARC, ^+^ HPF and DC show similar radiographic features with different histology, and EC is peripheral DC.

**Table 2:** Histological feature of odontogenic cyst based the 5th edition of WHO [2].

Inflammatory Cysts	Essential Histological Features
Radicular cyst (Apical)	The cyst is lined by non-keratinized stratified squamous epithelium exhibiting plexiform proliferation, exocytosis, and spongiosis. The cyst wall consists of fibrovascular tissue with a variable amount of inflammation.
Residual Cyst	
Inflammatory collateral cyst	
**Developmental cysts**	
Dentigerous cyst	The cyst is lined by uniformly thin non-keratinized stratified squamous epithelium, and the cyst wall consists of delicately collagenous stroma.
Hyperplastic dental follicle	The mass consists mainly of delicately collagenous stroma with occasional odontogenic rests. Occasionally, reduced enamel epithelium is present.
Eruption cyst	The cyst is lined by uniformly thin non-keratinized stratified squamous epithelium within the lamina propria.
Odontogenic keratocyst	The cyst is lined by uniformly thin parakeratinzed keratinized stratified squamous epithelium with a corrugated surface and hyperchromatic palisaded nuclei of the basal cell layer. The cyst wall consists of a fibrovascular stroma. Keratin may be present the cyst lumen.
Orthokeratinized odontogenic cyst	The cyst is lined by uniformly thin orthokeratinized keratinized stratified squamous epithelium with a flat, non-descriptive basal cell layer. Keratin may be present in the cyst lumen.
Glandular odontogenic cyst	The cyst usually shows multiple compartments and is lined by squamous or cuboidal epithelium with variable thickness. The most essential criterion is the presence of abluminal hobnail cells. The cyst lining may show cilia, microcysts, mucous cells, clear cells, papillary tufting, apocrine snouting and epithelial plaques.
Calcifying odontogenic cyst	The cyst is lined by ameloblastic epithelium with a variable amount of ghost cells which may undergo dystrophic calcification. The cyst wall consists of fibrovascular tissue with occasional subepithelial dentinoid deposition.
Lateral periodontal cyst and Botryoid odontogenic cyst	The cyst is lined by uniformly thin flat cuboidal cells with multiple epithelial plaques and clear cells. The cyst wall consists of fibrovascular tissueBotryoid odontogenic cyst shows multiple compartments with a similar cystic lining of the lateral periodontal cyst.
Gingival cyst of adult	The cyst is lined by uniformly thin flat cuboidal cells with multiple epithelial plaques and clear cells in the lamina propria.

**Table 3:** Summary of demographic in three different centers.

	All Three lefts	KAMC	KFMC	PSMMC	*p*-Value
Cases	372	141	82	149	
Age Mean (SD)	30 (16.6)	31 (15.9)	29.9 (17.4)	34.3 (16.6)	0.1002
**Gender** Male	225	83	44	98	
Female	147	58	38	51	
Male: Female ratio	1.53:1	1.43:1	1.15:1	1.92:1	
Mandible: Maxilla ratio	1.1:1	1.2:1	1.1:1	1.1:1	

**Table 4:** Summary of the clinicopathological features of each odontogenic cyst.

	Number of Case	Age Mean (SD)	Gender		Location	
			M	F	Mandible	Maxilla
**Inflammatory Cysts**						
Apical radicular cyst	186 (50.0%)	34.2 (14.8)	102 (54.8%)	84 (45.2%)	101 (54.31%)	85 (45.69%)
Residual cyst	4 (1.1%)	43.2 (10.2)	3 (75.0%)	1 (25.0%)	2 (50.0%)	2 (50.0%)
Inflammatory collateral cyst	1 (0.3%)	9 (NA)	1(100%)	0 (0.0%)	1 (100%)	0
**Developmental cysts**						
Dentigerous cyst	109 (29.3%)	26 (15.2)	72 (66.1%)	37 (33.9%)	70 (64.2%)	39 (35.8%)
Hyperplastic dental follicle	4 (1.1%)	12.5 (4.5)	1 (25.0%)	3 (75.0%)	2 (50.0%)	2 (50.0%)
Eruption cyst	2 (0.5%)	2	1 (50.0%)	1 (50.0%)	2 (100%)	0 (0%)
Odontogenic keratocyst	53 (14.2%)	34.6 (20.1)	38 (71.7%)	15(28.3%)	39 (73.6%)	14 (26.4%)
Orthokeratinized odontogenic cyst	2 (0.5%)	39.5 (20.5)	0 (0%)	2 (100%)	2 (100%)	0
Glandular odontogenic cyst	8 (2.1%)	40.75 (10.2)	4(50.0%)	4(50.0%)	1 (12.5%)	7 (87.5%)
Lateral periodontal cyst	3 (0.9%)	49.3 (12.7)	3 (100%)	0	2 (66.7%)	1 (33.3%)
Total	372		225	147	196	179

NA; Not Applicable.

**Table 5:** Summary of the treatment modalities of each odontogenic cyst.

	Total Cases	Treatment				
		Enucleation without Curettage	Enucleation with Curettage	Excision	Marsupialization	Resection
**Inflammatory cysts**						
Apical radicular cyst	172 (48.9%)	97 (56.4%)	12 (7%)	61 (35.5%)	2 (1.2%)	0 (0%)
Residual cyst	4 (1.1%)	2 (50.0%)	1 (25.0%)	1 (25.0%)	0 (0%)	0 (0%)
Inflammatory collateral cyst	1 (0.3%)	0 (0%)	0 (0%)	0 (0%)	1 (100%)	0 (0%)
**Developmental cysts**						
Dentigerous cyst	106 (30.1%)	64 (60.4%)	6 (5.7%)	34 (32.1%)	2 (1.9%)	0 (0%)
Hyperplastic dental follicle	4 (1.1%)	0 (0%)	0 (0%)	4 (100%)	0 (0%)	0 (0%)
Eruption cyst	1 (0.3%)	0 (0%)	0 (0%)	1 (100%)	0 (0%)	0 (0%)
Odontogenic keratocyst	52 (14.8%)	20 (38.5%)	12 (23.1%)	17 (23.7%)	1 (1.9%)	2 (3.8%)
Orthokeratinized odontogenic cyst	2 (0.6%)	0 (0%)	1 (50.0%	1 (50.0%)	0 (0%)	0 (0%)
Glandular odontogenic cyst	8 (2.2%)	2 (25.0%)	2 (25.0%)	3 (37.5%)	0 (0%)	1 (12.5%)
Lateral periodontal cyst	2 (0.6%)	1 (50.0%)	0 (0%)	1 (50.0%)	0 (0%)	0 (0%)
**Total**	352	186 (52.8%)	34 (9.6%)	123 (35.0%)	6 (1.7%)	3 (0.9%)

**Table 6:** Summary of the Treatment modalities of each odontogenic cysts.

	Number of Cases	Recurrence	No Recurrence	Follow-Up Period
Apical radicular cyst	159	1 (0.62%)	158 (99.8%)	6 months–Three years
Dentigerous cyst	92	4(4.3%)	88 (95.7%)	One year–Seven years
Odontogenic keratocyst	47	8 (17.0%)	39 (83.0%)	One year–Two years
**Total**	298	13 (4.4%)	285 (95.6%)	N/A

**Table 7:** Comparison of OC distribution in the current study and other Saudi and gulf countries.

	Current study (Three Centers), Riyadh, SA	Al-Rawi et al., Tawam Hospital, Abu Dhabi, UAE [10]	Alsheddi et al., King Saud University, Riyadh, KSA [9]	Ali MA et al., Kuwait University, Jabriya, Kuwait [11]
Sample size	372	121	470	200
Period	12 years	20 years	26 years	5 years
Mean Age	32 years	NR	30 years	NR
Male: Female ratio	1.53:1	0.86:1	1.4:1	1.5:1
Mandible: Maxilla ratio	1.1:1	1.3:1	1.1:1	1.7:1
**Inflammatory cysts**				
Apical radicular cyst	186 (50.0%)	96 (79.3%)	302 (54.9%)	95 (47.5%)
Residual cyst	4 (1.1%)	NR	31 (5.6%)	8 (4.0%)
Inflammatory collateral cyst	1 (0.3%)	NR	5 (0.9%)	NR
**Developmental cysts**				
Dentigerous cyst	109 (29.3%)	17 (14.0%)	118(21.5%)	51(25.5%)
Eruption cyst	2 (0.5%)	NR	1 (0.2%)	NR
Odontogenic keratocyst	53 (14.2%)	NR	69 (12.6%)	30 (15.5%)
Orthokeratinized odontogenic cyst	2 (0.54%)	NR	7 (1.3%)	3 (1.5%)
Glandular odontogenic cyst	8 (2.1%)	NR	5 (0.9%)	3 (1.5%)
Calcifying odontogenic cyst	NR	NR	11(2.0%)	4 (2.0%)
Lateral periodontal cyst	3 (0.8%)	8 (6.6%)	NR	NR
Gingival cyst of adult	NR	NR	1 (0.2%)	NR
**Other cysts**				
Odontogenic cyst, NOS	4 (1.1%)	NR	NR	6 (3.0%)

NR; Not Reported.

**Table 8:** Comparison of OC distribution in the current study and selected international studies.

	Current study (Three Centers), Riyadh, SA	Meningaud et al., Pitié-Salpêtrière University Hospital, Paris, France [14]	Ochsenius et al., University of Chile Chile [12]	Açikgöz et al., Ondokuz Mayis University Turkey [15]	Daley et al., University of Western Ontario, Canada [16]
Sample size	372	695	2944	452	6847
Period	12 years	10 years	28 years	9 years	26 years
Mean age	32 years	41.8	NR	NR	NR
Male: Female ratio	1.53:1	1.86:1	1.1:1	1:1.1	NR
Mandible: Maxilla ratio	1.1:1	3:1	0.67:1	1:1	NR
**Inflammatory cysts**					
Apical radicular cyst	186 (50%)	372 (53.5%%)	1494 (50.7%)	251 (54.7%)	4468 (65.15%)
Residual cysts	4 (1.1%)	32 (4.6%)	328 (11.2%)	NR	NR
Inflammatory collateral cyst	1 (0.3%)	NR	113 (3.8%)	NR	33 (0.48%)
**Developmental cysts**					
Dentigerous cyst	109 (29.3%)	154 (22.3%)	546(18.5%)	122 (26.6%)	1662 (24.08%)
Eruption cysts	2 (0.5%)	NR	NR	NR	40 (0.58%)
Odontogenic keratocyst	53 (14.2%)	133 (19.1%)	421 (14.3%)	15 (3.3%)	335 (4.88%)
Orthokeratinized odontogenic cyst	2 (0.54%)	NR	NR	NR	NR
Glandular odontogenic cyst	8 (2.1%)	2 (0.2%%)	NR	NR	6 (0.04%)
Calcifying odontogenic cyst	NR	NR	NR	NR	18 (4.59%)
Lateral periodontal cyst and Botryoid odontogenic cyst	3 (0.8%)	2 (0.2%)	17 (0.6%)	1(0.2%)	106 (1.48%)
Gingival cyst of adult	NR	NR	10 (0.3%)	NR	33 (0.48%)
**Other cysts**					
Odontogenic cyst, NOS	4 (1.1%)	NR	NR	NR	NR

## Data Availability

The data presented in this study are available on request from the corresponding author. The data are not publicly available due to the hospital policies.
